# Designing Brains for Pain: Human to Mollusc

**DOI:** 10.3389/fphys.2018.01027

**Published:** 2018-08-02

**Authors:** Brian Key, Deborah Brown

**Affiliations:** ^1^School of Biomedical Sciences, University of Queensland, Brisbane, QLD, Australia; ^2^School of Historical and Philosophical Inquiry, University of Queensland, Brisbane, QLD, Australia

**Keywords:** pain, consciousness, feeling, noxious stimuli, cortex, awareness, qualia

## Abstract

There is compelling evidence that the “what it feels like” subjective experience of sensory stimuli arises in the cerebral cortex in both humans as well as mammalian experimental animal models. Humans are alone in their ability to verbally communicate their experience of the external environment. In other species, sensory awareness is extrapolated on the basis of behavioral indicators. For instance, cephalopods have been claimed to be sentient on the basis of their complex behavior and anecdotal reports of human-like intelligence. We have interrogated the findings of avoidance learning behavioral paradigms and classical brain lesion studies and conclude that there is no evidence for cephalopods feeling pain. This analysis highlighted the questionable nature of anthropometric assumptions about sensory experience with increased phylogenetic distance from humans. We contend that understanding whether invertebrates such as molluscs are sentient should first begin with defining the computational processes and neural circuitries underpinning subjective awareness. Using fundamental design principles, we advance the notion that subjective awareness is dependent on observer neural networks (networks that in some sense introspect the neural processing generating neural representations of sensory stimuli). This introspective process allows the observer network to create an internal model that predicts the neural processing taking place in the network being surveyed. Predictions arising from the internal model form the basis of a rudimentary form of awareness. We develop an algorithm built on parallel observer networks that generates multiple levels of sensory awareness. A network of cortical regions in the human brain has the appropriate functional properties and neural interconnectivity that is consistent with the predicted circuitry of the algorithm generating pain awareness. By contrast, the cephalopod brain lacks the necessary neural circuitry to implement such an algorithm. In conclusion, we find no compelling behavioral, functional, or neuroanatomical evidence to indicate that cephalopods feel pain.

## Introduction

Why has the question of whether and which animals experience pain become so vexed? Among research topics, consciousness is unique in being private, first-personal and subjectively known. This makes theorizing about it a “hard problem” (Chalmers, 1995) because the subjective nature of feelings can only be definitively known by first-person experience and verbal report, i.e., by those creatures capable of both thought and language, namely, humans. As yet, there is no clear understanding of how feelings emerge from organic tissue. How is it that firing of nerve impulses in the human brain can generate either pain or pleasure or alternatively remain non-conscious? In the absence of both verbal reports and a neurobiological basis of feelings, researchers tend to rely overly on behavioral observations and “benefit of the doubt" assumptions (i.e., the precautionary principle) to decide whether certain species of animals are capable of feeling.

The classical approach to determining whether an animal feels pain is to observe its behavioral response to a noxious (harmful) stimulus. Behavioral studies are based on the premise that the behavior reflects some qualitative feature of the experience (e.g., an avoidance response reflects unpleasantness). The difficulty here of course is distinguishing whether the behavior truly demonstrates an underlying experience of pain. Analyses can be supported by ablation studies that remove portions of the nervous system that are believed to be involved in conscious rather than non-conscious behaviors. This approach then becomes laden with assumptions about which neural regions are involved in conscious behaviors in humans and whether these same regions and their functions are phylogenetically conserved.

To begin to address the question of whether an animal can, or cannot, experience pain requires a working definition of pain that is broadly applicable across phylogenetically distant species. We simply describe pain here as an unpleasant feeling. This definition indicates that a feeling that is not unpleasant is not pain. Consequently lobotomized patients who claim that they are experiencing pain that is not unpleasant (Bain, 2014) cannot therefore be experiencing pain. Because pain is a feeling, it is then not possible to have an unfelt pain as some have argued (Palmer, 1975). Agreeing on what a “feeling” is has been notoriously difficult (Searle, 2000; Carruthers and Schier, 2017). Feelings have been variously referred to as “conscious awareness,” “inner awareness,” (Farrell and McClelland, 2017) “subjective experience,” (Tye, 1986; Sytsma and Machery, 2010) “something-it-is-like” for the subject (Nagel, 1974), sentience (Harnad, 2016), “phenomenal consciousness” (Block, 1995) and “qualia” (Tye, 1994).

It is widely acknowledged that feelings share a close relationship with awareness (Natsoulas, 1983, 1999; Berger, 2014; Carruthers, 2015; McClelland, 2015; Farrell and McClelland, 2017; Kouider and Faivre, 2017; LeDoux and Brown, 2017). Given that awareness in any system is dependent on detection of change in the state of the system, then a brain must be able to selectively monitor internal changes in its neural information processing in order to be subsequently aware of them. Feelings, however, are more than detection of state change – there needs to be some implicit knowledge (Schacter, 1992) about the nature of what the brain is currently processing (Cleeremans, 2011). How does a creature generate such implicit understanding? If the internal monitor was a model of sensory processing that could accurately predict the future state of the processing, then that model would possess implicit knowledge or understanding of its internal operations. By way of analogy, if an artificial neural network was trained to predict the outcome of a chess match between two chess champions on the basis of the opening sequence of moves it would then possess some implicit knowledge (contained within the synaptic weights and connectivity of the network) of the players strategies. In comparison, a naïve observer network that merely monitored the game so as to report the outcome would lack any awareness of player strategy.

Returning to an animal nervous system, if a model network was monitoring sensory processing arising from a noxious stimulus then it would contain implicit knowledge about the type of stimulus (e.g., burning, freezing, stabbing, or cutting) as well as its intensity and location. Thus, establishing whether an animal’s nervous system has the capacity to observe and predict an outcome of its sensory processing following a noxious stimulus is a reasonable test of the animal’s capacity to feel pain. This strategy is not burdened by any need to explain the hard problem of how a conscious experience might be expected to feel qualitatively. We contend that an internal model of sensory processing is necessary for implicit awareness but not sufficient for the explicit qualitative feeling of pain. While the necessity of predictive modeling of sensory processing following a noxious stimulus is a significant first hurdle in assessing whether an animal is considered at least a possible candidate for experiencing pain, it is considerably less stringent then requiring an understanding of how the nervous system generates the qualitative nature of the pain experience itself.

Recently it has been claimed that some species of mollusca can experience pain (Mather, 2008, 2016; Mather and Carere, 2016; Godfrey-Smith, 2017). In the following sections, we briefly describe the molluscan nervous system before critically evaluating evidence purportedly supporting feeling in these creatures. This analysis will reveal that molluscs clearly exhibit non-conscious nocifensive behaviors in response to certain types of noxious stimuli. However, behavioral studies have been found wanting with regards to pain in molluscs. In an attempt to move away from weak inferences about pain based on behavioral studies we instead adopt the necessity test for animal pain based on a neuroanatomical framework containing model prediction networks. After discussing this framework in detail, we conclude that molluscs are incapable of feeling pain since the nervous system of molluscs (unlike humans) lacks the neural architecture required to implement the requisite computations defined within this framework.

## Mollusc Nervous System

Mollusca consist of over 74,000 species that inhabit marine, freshwater, and terrestrial environments (Dunn et al., 2014). They have diverse body plans and encompass bivalvia, gastropods, and cephalopods which include animals such as clams, mussels, snails, squid, and octopi. The most basal lineages in this group possess two bilateral symmetrical longitudinal nerve cords embedded in a plexus of neural cell bodies, that coalesce and form a ring in the head (Faller et al., 2012). This nerve ring is referred to as the brain and contains an uncompartmentalized neuropil surrounded by neuronal perikarya. In more differentiated nervous systems, neurons cluster and form distinct ganglia interconnected by nerve fibers called connectives. Cephalopods (nautilus, cuttlefish, squid, and octopus) have the most morphologically complex nervous system in mollusca (Budelmann, 1995) and also display remarkably sophisticated behavioral repertoires and cognition (Zarrella et al., 2015; Mather and Dickel, 2017). Given these properties and recent claims that cephalopods are the best candidates for invertebrate consciousness (Mather, 2008; Mather and Carere, 2016), we have restricted our discussion regarding sensory awareness to this group and only to those few (of ∼700) species (Kröger et al., 2011) that have been experimentally investigated.

**FIGURE 1:**
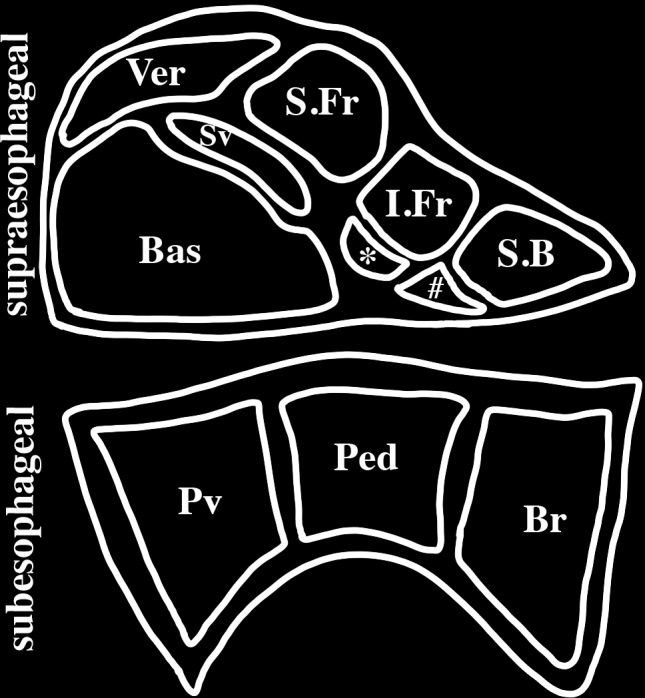
Schematic representation of a mid-sagittal section of an octopus brain. The brain is encased in a cartilaginous capsule and consists of two principal regions: the supraesophageal region lying above the esophagus and the subesophageal region lying below the esophagus. The principal lobes of the midline supraesophageal region consists of the vertical (V), subvertical (Sv), superior frontal (S.Fr), inferior frontal (I.frontal), superior buccal (S.B), posterior buccal (#), subfrontal (^∗^) and basal (Bas) lobes. The inferior frontal consists of a median lobe (shown here) and a lateral lobe (not shown here) that is located lateral to the midline. The subesophageal brain is partitioned into three lobes: brachial (Br) which gives rise to the brachial nerves to the arms, pedal (Ped) and palliovisceral (Pv) lobes.

The octopus nervous system is partitioned into three principal regions: the brain (40 million neurons within a cartilaginous capsule); optic lobes beneath the eyes (130 million neurons) and the associated small peduncle and olfactory lobes; and the peripheral ganglia of the arms (350 million neurons) (Young, 1963). The arm nervous system consists of both sensory and motor neurons controlling simple movements that contribute to goal directed behaviors even when the arm is severed from the body (Sumbre et al., 2001). The brain consists of two principal regions: a supraesophageal complex of lobes (enlarged ganglia), which lie above the esophagus, and a subesophageal complex, which lies below the esophagus (**Figure 1**). Together these regions consist of about 25 major lobes with each comprising an outer layer of neuronal cell bodies and an inner neuropil. The axon connectives between these lobes are short and contribute to making the lobes appear fused as a single large mass.

The cephalopod nervous system, like that of vertebrates, is hierarchically organized into levels that sequentially control behaviors (Boycott, 1961; Young, 1976, 1988; Sumbre et al., 2001; Zullo et al., 2009; Zullo and Hochner, 2011; Kobayashi et al., 2013). Sensory and memory brain centers such as the optic lobes for vision, the inferior frontal lobes for tactile discrimination and the vertical and median superior frontal lobes for memory and learning are important in regulating elaborate behaviors such as camouflage patterning, navigation, attack and evasive planning (Mather and Dickel, 2017). Each of these brain centers project directly or indirectly to the higher motor centers located in the basal and peduncle lobes (Ba, **Figure 1**). The higher-order centers coordinate complex motor action like swimming and walking. Following ablation of the higher-order motor centers octopi are no longer able to perform spontaneous movements. The higher motor centers project to and regulate the intermediate and lower motor centers (in the subesophageal region). The intermediate motor centers coordinate simple movements such as arms working in synchrony. The intermediate motor centers control lower motor centers (which are motor neuron clusters present in both the subesophageal region and arms). The lower motor centers regulate select muscle groups such as those involved in eye and single arm movements.

## Brain Regions Responsible for Behavioral Responses to Noxious Stimuli in Mollusca

Octopus blinded by sectioning of the optic nerves can use tactile information arising from a single arm to discriminate between two texturally distinct objects (Wells, 1964). This tactile discrimination is achieved through a reward and punishment training regime involving a “positive” object whose selection is rewarded by food (i.e., sardine), and a “negative” object, whose selection is punished with a mild electric shock. This shock also elicits an escape response involving the animal swimming away to another place in the aquarium (Wells, 1959b). Within a few trials, the negative object is pushed away or rejected while the positive object is accepted and passed toward the mouth. Such learning behavior is commonly interpreted as evidence that the animal consciously feels pain following electric shock (Andrews et al., 2013). If this premise is true, then it should be possible to localize the site of pain in the octopus brain by assessing the effects of specific brain lobe ablations on the performance of tactile discrimination during operant conditioning.

Removal of the entire supraesophageal brain completely destroys the ability of octopus to learn to reject objects associated with electric shock (Wells, 1959a). When presented with negative objects these brain-ablated individuals repetitively accept them despite the shock punishment. This result seems consistent with the idea that pain is generated in this part of the brain. If the inferior frontal system (posterior buccal, inferior frontal, and subfrontal lobes) are selectively spared from the surgical ablation of the supraesophageal brain, animals regain their ability to learn to discriminate. Accordingly “pain” must be arising somewhere in this brain region.

However, the gross motor behavior of these brain-ablated animals is severely compromised. Animals can no longer walk or swim and instead sit on the bottom of the holding tank with arms in disarray (Wells, 1959a). By instead selectively removing only the inferior frontal system, while leaving the rest of the supraesophageal brain intact, animals display normal gross motor behaviors (Wells, 1978). In the absence of these lobes animals do not reject negative objects. Although these confirmatory results support the idea that “pain” arises in this very specific region of the supraesophageal brain, these animals now strongly react to electric shock. After receiving an electric shock for failing to reject the negative object, an animal lacking the inferior frontal system rapidly swims away while dragging the tightly grasped object in its arm (Wells, 1961). This escape behavior demonstrates that the animal is capable of responding to an electric shock (supposedly it can still feel “pain”) and yet it doesn’t release the object. These results expose a dissociation between learning and any so-called “pain” felt by the animal. Thus “pain” is not the driver for octopus learning to respond to negative objects. Avoidance learning is therefore not evidence of pain. Rather, these results are consistent with the inferior frontal system directly regulating arm motor behaviors. When present, the inferior frontal system activates a reject motor program (or inhibits an accept motor program) in response to a noxious stimulus. When ablated, the reject response is not activated (or the accept program is inhibited) and a default accept program dominates.

It could be argued that “pain” was generated in the brachial lobe of the subesophageal brain (an arm motor center) and then relayed to the inferior frontal system where it regulated learning. Thus, when only the inferior frontal system was spared ablation in the supraesophageal brain, “pain” could still drive learning since it arose from the lower subesophageal brain. However, while afferent sensory fibers arising from the arms project via the brachial nerves and innervate the brachial lobe of the subesophageal brain, they terminate on motoneurons (Young, 1978). No second-order sensory fibers subsequently project from the brachial lobe to the inferior frontal system. Rather, sensory afferents from the arm enter the cerebrobrachial connective and terminate directly in the inferior frontal system (Budelmann and Young, 1985). Thus “pain” is not generated in the subesophageal brain and is not relayed to the inferior frontal system.

**FIGURE 2:**
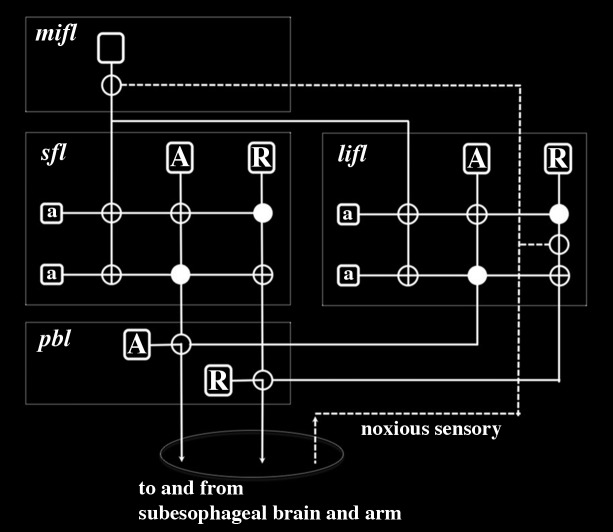
Wiring diagram for the inferior frontal system controlling tactile discrimination behaviors in Octopus as proposed by Young (1983, 1991). This representation neglects positive sensory input associated with acceptance behaviors and instead depicts input from noxious sensory fibers from the arms that enter the inferior frontal lobes from the cerebro-brachial connective (Budelmann and Young, 1985). The stylistic circuit is drawn to highlight similarities in neural connectivity as previously described for learning and memory circuits of the superior frontal and vertical lobes (Hochner and Shomrat, 2013; Shomrat et al., 2015). Large projection neurons are depicted as unfilled rounded rectangles and in the subfrontal lobe (*sfl*), lateral inferior frontal lobe (*lifl*) and posterior buccal lobe (pbl) there are two principal types – acceptance (A) and rejection (R) – which selectively project to one another. The projection neurons associated with tactile discrimination in the median inferior frontal lobe (*mifl*) project to both the *sfl* and *lifl* where they terminate on small amacrine interneurons (a). Although this projection neuron is represented as having axon branches this is simply diagrammatic and it is most likely that there are multiple types of these neurons that have different innervation densities in the *sfl* and *lifl*. Synapses are represented as filled circles (inhibitory) or unfilled circles (excitatory). Noxious sensory fibers selectively innervate projection neurons in the *lifl* and *mifl*. Electric shock would be expected to activate R projection neurons in the *lifl* and depending on their state of activation by local amacrine interneurons, these R neurons would activate R projection neurons in the *pbl* and lead to a reject behavior. Noxious sensory fibers projecting to the *mifl* would cause a cascade of modulatory activity in the *sfl* and *lifl* that would robustly regulate motor behavior. With tactile discrimination training (using either or both positive and negative reinforcements) this circuitry outlined here would enable behavioral learning and memory formation that could readily increase efficacy of motor actions.

To continue to accept that an octopus feels pain during operant conditioning it is necessary to suppose that this pain must be multiply realized throughout the brain. Multiple realization is the hypothesis that a mental state (e.g., pain) can occur in many different organisms with vastly different neural morphologies (such as humans and molluscs) (Kim, 1992). Here, we apply the term to include the possibility that pain would also need to arise in many different independent regions within the same nervous system in order to account for the ablation data in molluscs. To adhere to the idea that molluscs feel pain one needs to propose that pain is generated locally in the inferior frontal system and also in all other brain regions associated with behaviors elicited by noxious stimuli. Pain associated with an escape swim response must be generated outside of the inferior frontal system since this escape behavior continues in the absence of this system. Likewise pain from electric shocks used during operant conditioning involving visual stimuli must also be generated elsewhere since this learning occurs in the absence of the inferior frontal system (Wells, 1961). Since this visual learning is dependent on the superior frontal and vertical lobes (Boycott and Young, 1955) pain must also be generated locally within these lobes. However, multiple realization of pain in the inferior frontal system and the vertical-superior frontal lobes is unlikely given that the known circuitry in these regions specifically supports neural processing associated with learning and memory formation (**Figure 2**) (Young, 1991; Shomrat et al., 2015). These brain regions have wiring patterns that share strong structural and functional similarities with the human hippocampus (Young, 1991; Shomrat et al., 2015). While the hippocampus is important for learning and memory involving pain in humans, it is not involved in the neural computations proposed to underlie the sensation of pain (Bastuji et al., 2016; Tseng et al., 2017; Garcia-Larrea and Bastuji, 2018). This neural processing underlying pain in humans will be examined in more detail below.

This discussion started with the premise that octopus feels pain. We have now shown that this assumption creates some conceptual difficulties and leads to the conclusion that discrete isolated regions of the brain such as the inferior frontal system generate pain. Interestingly, the median inferior frontal lobe within the inferior frontal system is not essential for tactile learning during operant conditioning (Wells, 1959a; Wells and Young, 1975; Wells, 1978). While selective removal of the subfrontal lobe leads to markedly reduced tactile discrimination, it is still possible to produce some learning using extended reward and punishment training (Wells and Young, 1975; Wells, 1978). This residual learning in response to electric shock punishment can now only be achieved by circuitry in the remaining undamaged lateral inferior frontal lobes within the inferior frontal system. The simple circuitry in this lobe is similar in principal to that underlying classical conditioning of gill and siphon withdrawal reflexes in Aplysia in response to electric shock (Kupfermann et al., 1974; Carew et al., 1983; Benjamin and Kemenes, 2008; Hochner and Glanzman, 2016). As noted above it does not resemble the global, integrative neural network matrices considered to generate pain in vertebrates (Garcia-Larrea and Peyron, 2013; Garcia-Larrea and Bastuji, 2018). Rather than pain driving tactile discrimination learning in octopus, the data are more consistent with noxious sensory information autonomously regulating local neural circuits at multiple hierarchical levels in the octopus nervous system. This conclusion is further supported by the isolated arm experiments described below.

Altman (1971) revealed that isolated amputated arms of octopus are able to either accept a sardine or reject a sardine soaked in noxious quinine hydrochloride. Thus, accept and reject responses are reflex behaviors generated by local sensory and motor circuitry in the arm and are not contingent upon the animal consciously feeling pain. However, Altman (1971) showed that different levels of the brain exhibited hierarchical control of the reject reflex. When only the supraesophageal brain was removed (leaving only the subesophageal brain intact), animals could no longer reject objects. The reflex was regained when the inferior frontal system was spared from this ablation. This result revealed that the subesophageal brain inhibited the reflex while the inferior frontal system facilitated this reflex. Recent isolated octopus arm experiments have further demonstrated a classic withdrawal reflex response of arms to either pinches or noxious chemical applied to the tip of the arm (Hague et al., 2013). The reject arm reflex in octopus has some similarity to the spinal control of leg withdrawal reflexes of humans in response to peripheral noxious stimuli (Hagbarth, 1960). Taken together, the brain lesion experiments and the isolated arm preparations reveal that nocifensive behavior in response to noxious stimuli are stereotyped movements executed principally by local arm circuitry but regulated hierarchically in the brain and as such provide no evidence that octopi feel pain.

Arm injury in some octopi causes arm autotomy distal to the injury site. Following autotomy, animals initially display wound grooming followed by guarding behavior where the injured limb is shielded by other arms (Alupay et al., 2014). This behavior is accompanied by reduced local mechanosensory threshold for arm withdrawal and escape responses. Removal of all the supraesophageal brain except for some optic lobe stalk and partial basal lobes (containing the higher motor centers) did not abolish the grooming and guarding behaviors. These behaviors were only lost with complete supraesophageal brain removal, which is consistent with the known role of basal lobes in controlling general body movement (Wells, 1959a). These results further demonstrate the autonomous nature of the behavioral responses to short term noxious stimuli and chronic injury. These nocifensive behaviors do not provide any evidence that octopi feel pain and that pain is driving these motor actions (Crook and Walters, 2011; Crook et al., 2013; Butler-Struben et al., 2018).

Such behaviors are instead adequately accounted for by non-conscious, feedforward neural circuits executing hierarchically controlled motor actions (Hochner and Shomrat, 2013; Shomrat et al., 2015; Hochner and Glanzman, 2016; Levy and Hochner, 2017). Numerous studies indicate that goal-directed movements are predominantly under control of autonomous motor programs in the peripheral nervous system and that the central brain is involved in activating these programs (Sumbre et al., 2001, 2006; Levy et al., 2015; Levy and Hochner, 2017). The ability of complex behaviors to be executed using non-conscious hierarchical control systems in octopus has been convincingly demonstrated by progressively increasing external microstimulation of the basal motor lobes (Zullo et al., 2009). A variety of elementary motor actions can be recruited in various combinations leading to the production of complex behavioral responses as a result of simply increasing electrical stimulation to these lobes.

## Behavior is Not Sufficient to Infer Conscious Awareness

Despite known difficulties in inferring sentience from behavior, stimulus-response paradigms continue to be widely used in animal studies to assess the presence of feelings such as pain (King and Porreca, 2014; Barrett, 2015). This is particularly problematic in molluscs when the anatomy and physiology are so divergent from mammals (Crook and Walters, 2011). A clear distinction needs to be drawn between nociception and pain (Crook and Walters, 2011) and importantly, nociception in molluscs should not be confused with evidence for pain-like states (Crook et al., 2013). Similar arguments have been countenanced for insects (Adamo, 2016a,b).

While many animal studies still rely on non-conscious action responses, others have embraced an idea that complex behavior involving goal pursuit is a better indicator of conscious awareness. However, the obligatory association between goal pursuit and conscious processing is challenged even in humans (Custers and Aarts, 2010). Many complex and goal-oriented behaviors, such as the *Drosophila* male courtship ritual, can be deconstructed into a series of innately driven and genetically determined stereotyped subroutines (Manoli et al., 2006). Awareness of a goal or the presence of feelings clearly plays no role in the courtship ritual, since this complex behavior can be performed by headless flies (Pan et al., 2011). There is no evidence that complex learning in insects involves sentience (Giurfa, 2013; Chittka, 2017). There is no need to assume conscious awareness in either insects or molluscs in order to explain complex behaviors when non-conscious neural networks can effectively account for such abilities (Ardin et al., 2016; Faghihi et al., 2017; Goldschmidt et al., 2017; Müller et al., 2017; Peng and Chittka, 2017; Perry et al., 2017; Roper et al., 2017).

Given the specious relationship between complex behaviors and conscious awareness, there is some support for the idea that “flexible behavior” (i.e., the ability of an animal to adapt its behavior in response to changing environments or novel challenges; Griffin, 1976) is a better indicator of conscious awareness (Bekoff, 2003; Edelman and Seth, 2009; Seth, 2009; Droege and Braithwaite, 2014; Mather and Carere, 2016). However, conflating flexible behavior with feelings remains problematic, since even innate, stereotyped behaviors are known to exhibit considerable plasticity. For instance, spinal central pattern generators (CPGs) controlling limb movements during vertebrate locomotion (Frigon, 2017) can easily adapt to changing environments to allow an animal to locomote in both water (swimming) and on land (stepping) using vastly different gait kinematics (Ryczko et al., 2015). The non-conscious nature of this flexible motor behavior is supported by evidence that distinct, behavior-specific CPG outputs can be achieved even in the isolated vertebrate spinal cord. The autonomous decentralized and flexible nature of the CPG is exemplified in the millipede, which is able to regulate kinematics of each leg in response to local environmental cues (Kano et al., 2017).

It has been suggested that some animals (e.g., fish) are sentient because they appear to display declarative memory, conditioned place preference, trace conditioning and transitive inference. However, none of these behaviors necessarily rely on subjective awareness (Reber et al., 2012; Mudrik et al., 2014); and so embracing these criteria will lead to erroneous inferences concerning sentience (Key, 2015, 2016). In summary, relying on behavior alone is not sufficient to justify claims of conscious awareness in an animal.

Despite inherent problems with using behavior as a yardstick for consciousness it has been argued that cephalopods possess a simple form of consciousness referred to as “primary consciousness” (Mather, 2008). Mather (2008) seems to associate this type of consciousness with the ability of some cephalopods to display complex behaviors, to learn and to learn using simple concepts. While we have already dismissed complex and flexible behaviors as a measure of feeling, Mather’s adoption of learning and use of simple concepts as a measure of primary consciousness is mistaken given that such behaviors could be either implicit or explicit (Schacter, 1992), and only the latter could be argued to depend upon the availability of concepts. In many instances, anthropomorphic claims are used to defend conscious awareness in cephalopods. For instance, Mather (2016) claims that octopi adopt “cautious” approaches to stinging sea anemones and even blow jets of water at the anemone and hence do not just respond reflexively to noxious stimuli. These anthropomorphic descriptions based on anecdotal observations need to be critically assessed within the context of innate behaviors and implicit learning (LeDoux and Daw, 2018).

Mather (2008) suggests that play behavior exhibited by octopi is consistent with these animals having consciousness. Mather defines behavior as play-like if any of the following actions were performed with novel plastic objects: pushing or pulling of the object in one coherent action; dragging an object by an arm across the surface of the water in more than one direction; or passing the object between the arms more than six times (Kuba et al., 2006). Using these criteria 9/14 octopi in her study were reported to engage in play-like behavior. While no evidence is provided that such behavior actually represents any form of play there is the underlying assumption that it involves conscious awareness of “fun” since it is labeled as “play-like.” However, recent optogenetic experiments in mice have revealed that craving, selective attention and so called play-like activity toward novel objects is automatically induced by simply activating a single neural pathway between the medial preoptic area and the ventral periaqueductal gray area (Park et al., 2018). The take-home lesson here is that causes of behavior may not be extrapolated from observation of the behavior alone and that describing animal behavior (e.g., as play-like) based on anthropometric measures is question-begging.

Bronfman et al. (2016) have proposed that animals capable of specific types of associative learning (referred to as “unlimited associative learning”) must be sentient. Unlimited associative learning is considered to involve complex behaviors rather than simpler forms of associative learning. This hypothesis is again built on the false premise that complex behavior is dependent on sentience. For instance, Bronfman et al. (2016) consider that an animal can feel if it is capable of learning to associate an object by a combination of its properties (e.g., color, shape, and texture) with the future presentation of food, whereas each property alone is not sufficient for eliciting a behavioral response. Unfortunately no evidence is provided that this form of associative learning necessarily involves sentience. Bronfman et al. (2016) refer to compound operant conditioning in octopus (as in Hochner and Shomrat, 2013) as evidence of sentience.

Hochner and Shomrat (2013) showed that octopus could be trained not to attack a red ball (containing the integrated properties of brightness and shape) by negative reinforcement with electric shocks. According to Bronfman et al. (2016) this learning was evidence of unlimited associative learning since the animals continued to approach balls (same shape) that were white instead of red. However, Shomrat et al. (2008) describe the neural circuitry underlying this associative learning and conclude that “our results fit a simple feed-forward model of octopus avoidance-learning systems.” There is no evidence, in short, that such behavior demands sentience (LeDoux and Daw, 2018). Of interest is recent research demonstrating that non-conscious (i.e., subliminal) sensory stimuli such as novel pairs of visual and spoken presentations of words can mediate complex associative learning in humans (Scott et al., 2018). This builds on earlier research demonstrating that awareness of conditioning stimuli is not needed for instrumental conditioning in humans (Pessiglione et al., 2008). Such findings argue against the necessity of sentience for unlimited associative learning in cephalopods.

## A Way Forward in Addressing Conscious Awareness

A foundational principle of evolutionary biology is that structure determines function. Call this the “SDF principle.” According to the SDF principle, the morphology of any biological tissue is the key to its physiological function. The structure of a nervous system imposes fundamental limitations of what it can and cannot do. For instance, the ability of an animal to perform non-gliding flight is determined by the structure (i.e., anatomy) of the animal’s wing or wing-like appendage. While the shape and form of these appendages varies considerably across winged species, there is a common design plan that enables the necessary aerodynamic force of lift to be generated (Lindhe Norberg, 2002). The anatomy of a wing explains how it can be used for flight. Consequently, any animal lacking the common design feature of the wing will lack the potential to perform non-gliding flight. Why suppose that the SDF principle does not also apply in explaining the capacity for feeling, that there is not some common design or structural features that explain the capacity for feeling across different species? It is this question that frames our current approach to the design of a nervous system that is capable of conscious awareness.

Once the properties of neural tissue deemed both necessary and sufficient for feeling sensory stimuli have been identified, then the assessment of whether any particular animal is likely or has the potential to feel or not can be reduced to the identification of those relevant properties in the animal’s nervous system. Given that there does not appear to be any solution to this problem in the near future, one way forward is to define the basic underlying design principles and use this knowledge to create a minimal neural architecture necessary (but not sufficient) to support pain. Two important questions provide a framework for addressing this problem. First, what sorts of algorithms need to be executed by a nervous system to generate pain? Second, how are those algorithms implemented in a nervous system? An answer(s) to the latter question would begin to expose some of the necessary neural architectural prerequisites for pain.

An argument against trying to identify the necessary neural architectures is that the solution to the algorithms may be multiply realized in different animals (Weiskopf, 2011). That is, different neural circuits may be able to implement the algorithms. This is not reason enough to disregard this approach since all that is needed is to identify all possible circuits in extant creatures. Given that this is likely to prove a formidable task, a better approach would be to define instead the generic architecture that enables multiple realization to be captured since multiple realization does not necessarily apply to basic computations (Keeley, 2000).

As proof of principle, we have tested this strategy by characterizing the necessary circuitry underlying rhythmic motor movements during locomotion of bilaterally symmetrical animals. The basic algorithm generating left-right rhythmical motor activity is an alternating left-right rhythmical muscle activity occurring at the same segmental or anterioposterior level. That is, there is sequential contraction and relaxation of the same muscles on the left and right sides of the body, respectively. If these muscles fail to exhibit this cyclic activity, then the animal no longer engages in left-right phased rhythmical locomotion. In order for left-right phase activity to be rhythmical, left muscles must be activated while those controlling the same muscles on the right must be simultaneously inhibited.

Given such an algorithm, what then is the circuitry that implements it? Reciprocal inhibition (i.e., activated neurons on one side cause muscle contraction and also inhibit the same muscles on the opposite side) is an essential component of left–right rhythmical locomotion since independent pacemakers on either side spontaneously drift in and out of phase (Friesen, 1995). While the specific interconnectivity of neurons (i.e., microcircuitry) that leads to reciprocal inhibition can be multiply realized between different species, all species possess neurons that project across the midline to reciprocally inhibit the other side so that left muscles are activated while right muscles are simultaneously inhibited. This crossed inhibitory circuitry involves activation and inhibition of excitatory motor neurons in almost all animal models, including: leeches, fish, and mammals. To date, only nematodes achieve simultaneous contraction and inhibition of muscles using a combination of both excitatory and inhibitory motor neurons (Wen et al., 2012). A left excitatory motor neuron activates right muscles and simultaneously excites a right inhibitory motor neuron that causes right muscles to relax. This crossed excitation of inhibitory motor neurons produces the alternating rhythmical muscle activity. Thus, by knowing whether an animal possesses the necessary neural architecture required to perform cyclic inhibition of left-right muscle activity (i.e., crossed connections that lead to simultaneous contraction and inhibition of the same muscles on left and right sides), it is possible to predict whether an animal is, at least, capable of performing locomotion based on left-right rhythmical contractions. If an animal lacks this fundamental neural architecture, then one can confidently conclude that it cannot perform this type of locomotion.

While we have concentrated on CPGs, there are numerous examples of conserved circuitry that subserve similar functions both within and across phyla (Loesel et al., 2013; Farris, 2015). For instance, basic circuitry for associative learning is conserved in the vertical lobe in octopi, mushroom bodies in insects and hippocampus in vertebrates (Katz, 2016). Olfactory glomerular-like structures are also involved in processing of olfactory sensory information in molluscs, insects and vertebrates (Strausfeld and Hildebrand, 1999; Eisthen, 2002; Farris, 2015). Likewise, the loss of either olfactory or visual neural circuitries within some species in a phyla correlates with the absence of behavioral responses to these sensory stimuli (Ramm and Scholtz, 2017). Interestingly, Scaros et al. (2018) have recently failed to morphologically identify olfactory glomeruli in embryonic and hatchling stages of development in the cuttlefish *Sepia officinalis*. However, olfactory glomeruli are not morphologically defined in developing late embryonic mice (Royal and Key, 1999). At this stage of development visualization of glomeruli formation relies on the expression of odorant receptor genes (Royal and Key, 1999). Similarly, olfactory glomeruli emerge slowly in developing *Xenopus* and never achieve the morphological definition of those in mammals (Byrd and Burd, 1991). Olfactory glomeruli are also not easily discernible by immunohistochemical staining in the adult frog *Rana catesbeiana* (Key and Akeson, 1990) and most glomeruli in adult zebrafish are anatomically indistinguishable (Braubach et al., 2012). The question as to whether *Sepia officinalis* possesses glomeruli and its implications for olfaction must await further more detailed investigation.

## A Neural Architecture Necessary for Feelings

**FIGURE 3:**
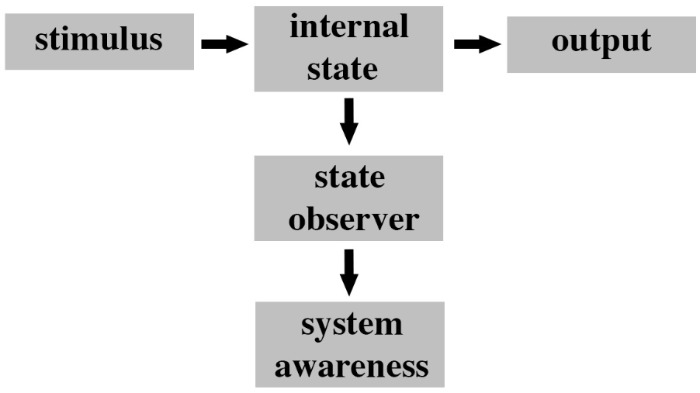
Fundamental organization of a system with awareness potency. The ground state of the system represents a stimulus response module whereby a stimulus (i.e., input) produces a change in the internal state of the system and this in turn leads to a specific output. In order for the system to begin to be aware of what it is doing there must be some independent process of observing changes in its own internal state. This process is performed by a state observer module that detects changes in the internal state that are responsible for the output. The state observer has a low level of awareness (e.g., a temperature gauge in a car engine is a state observer). An additional system awareness module needs to subsequently monitor changes in the state observer and to interpret this information in context of the overall functioning of the system. This second layer of monitoring represents a higher level of awareness (i.e., awareness of awareness). For instance, in the example of an engine temperature this module would detect rises in temperature and determine their significance to the car in respect to overall performance of the engine.

We hypothesize that one of the fundamental organizational principles of feeling nervous systems is that they must be able to internally monitor their own neural processing (i.e., internal states). Such internal monitoring is critical for any system to achieve a level of awareness of its own processes and to use that awareness to execute functions (Kwon and Choe, 2008; Jeremy, 2014). Air conditioning systems in buildings must monitor the internal temperatures of rooms in order to adjust air flow accordingly. Similarly, nervous systems must possess specialized neural circuitry to monitor their internal sensory processing of noxious stimuli in order to become aware and feel pain. We contend that there are at least three hierarchical levels of a system that are diagnostic for assessing whether that system has the potential to be aware. First, there must be a change in the internal state of the system (“internal state” in **Figure 3**) caused by the stimulus. This internal state is equivalent to the sensory processing pathways leading to some output (e.g., behavior). Second, the system needs to be able to monitor for changes in those internal states (“state observer” in **Figure 3**). Internal monitoring has a long history in consciousness studies (Lycan, 1995). Third, the system needs to become aware of those internal state changes (“system awareness” in **Figure 3**).

The role of awareness in consciousness and its independence from report and self-reflection is well debated in the literature (Farrell and McClelland, 2017). One of the design constraints of this framework is that the state observer and state awareness subsystems need to be external to (i.e., independent of) the sensory processing pathways (internal state) so that their processes do not mutually corrupt each other and to ensure that the prediction (i.e., awareness) is available for the function of the whole system (Cleeremans, 2011; Dehaene et al., 2017).

**FIGURE 4:**
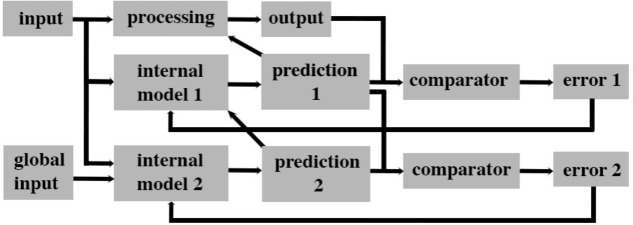
A parallel forward models algorithm that generates levels of awareness within a system. This scheme is based on the concept that a system that can accurately predict its future state based on its current state has awareness that is functionally significant for the system. This form of awareness is postulated to be necessary but not sufficient for subjective experiences such as pain. This algorithm processes input simultaneously along three parallel streams. The base level is the processing module that creates an output for the system. A second level consists of a module that creates a simplified model of the base level processing that predicts (predictions 1) the future output of the system. That prediction is then compared to the actual output in a separate module (comparator) and the error generated is used to adjust the internal model 1 to better predict future states (by attempting to reduce error 1 to zero). A second internal model (internal model 2) uses both the input to the system and available inputs from other systems to predict the outputs of internal model 1. Prediction 2 is feedback into internal model 1 to facilitate a rapid and accurate prediction 1. Prediction 1 likewise feeds back on to base level processing to control its output.

How could such an algorithm be implemented in an animal nervous system? We propose that when a system can predict the outcome of its current internal processes, then it must be capable of having a level of awareness of its internal state. Such predictions can be generated by an internal model of its processing that makes outcome predictions about ongoing neural activity. Modeling internal states and using the resultant predictions to rapidly adjust ongoing processing has been central to the field of plant engineering since the 1960s (Luenberger, 1971). For instance, the state of internal processes of a coal-fired power station can be indirectly monitored by a “state observer” (e.g., an artificial neural network; Paton, 2008) that continuously receives information about the current input (coal) and output (energy) of the station. The state observer is an “internal model” of the ongoing processing that makes predictions about a future output given a particular input. This prediction is then compared with the subsequent real output of the station and the error margin is used to adjust the processing of the coal and modify the state observer so that it becomes a more accurate predictor of the current processing state of the coal station (**Figure 4**).

The internal model (“internal model 1” in **Figure 4**) is a very efficient and rapid process because it does not need to monitor all the stages of processing in the plant. In plant engineering, human operators oversee the function of the internal model 1 and ensure overall fidelity of the system. In doing so, human operators also make use of information not available to the internal model (e.g., transport delivery systems, end-users, and global financial markets). This same algorithm can be applied to the processing of sensory information in a nervous system. In this case, human operators are replaced by a second level internal model (“internal model 2” in **Figure 4**). This second model and its prediction is equivalent to “system awareness” in **Figure 3**. It uses a copy of the input to a sensory system as well as “global input” (**Figure 4**) from other sensory systems to create its prediction. This second prediction (“prediction 2” in **Figure 4**) is a prediction of the output of the first internal model since it uses a comparator to determine the error (“error 2” in **Figure 4**) between prediction 2 and prediction 1. That error is then used to adjust the internal model 2 so as to reduce error 2. Predictions 1 and 2 both control (directly and indirectly, respectively) the ongoing processing so that the system produces an appropriate output.

Multiple internal models are fundamental building blocks of self-aware computing systems and computational “feelings” in agents (Lewis, 2016; Kounev et al., 2017; Lewis et al., 2017; Sánchez-Escribano, 2018). First-order internal models are restricted to monitoring specific processing events in order to efficiently control their behavior and to reduce overall processing time. Second-order internal models then correct errors in these first-order models arising from the noisy environment, track the consequences of the outcomes of the first-order models, and determine their relevance to and suitability for the whole functioning system. Second-order models enable the system to learn the consequences of its internal processing and to act for the benefit of the whole system. As such, they may be considered as displaying a rudimentary form of subjective awareness (i.e., they can predict the future on the basis of the system’s past experience via prediction error feedback). In the proposed algorithm, it is prediction 2’s (**Figure 4**) higher-level of awareness through its integration of global and local information that endows it with greater functional significance for the system, a proposed defining feature of conscious awareness (Dehaene et al., 2017). We argue that prediction 2 is a necessary (but not sufficient) condition for feelings. Even though such an “awareness” might become conscious, it is not sufficient to explain why it should feel like something, rather than nothing. As noted above, the aim here is not to provide a reductive analysis of what feeling is, but only to establish the legitimacy of demarcating boundaries between species of animals that are candidates for attributions of feeling and those that are not.

## Relationship to High-Order Theories of Awareness

Our algorithm (**Figure 4**) is distinct from Rosenthal’s higher-order theory of awareness (Rosenthal, 1993) in that we recognize the existence of multiple levels of awareness without asserting that awareness becomes immediately conscious through a higher-order representations of awareness. At present, our proposed algorithm is neutral on the necessity of higher-order thoughts. Its explanatory power can, however, be extended by exploring contributions made by other types of neural processing. Internal models are just one type of state observer (**Figure 3**). Working memory can temporarily store a copy of a sensory stimulus that can be compared subsequently with new incoming sensory information to assess changes in neural states. Such assessments are a form of internal monitoring and hence represent a level of awareness of internal state changes. Although we plan to examine how frameworks involving working memory could explain feeling states in future, this is a controversial topic given the importance of the prefrontal cortex to working memory and suggestions that this cortical region is not necessary for consciousness (Boly et al., 2017). How other types of memory (short-term, long term and associative), attention, ensemble coding, saliency, and executive control networks interact with and might further strengthen the framework also need to be explored.

## Relationship to Hierarchical Predictive Coding

We refer to our proposed framework as a parallel forward models algorithm (**Figure 4**). By definition forward internal models use inputs (e.g., sensory data) to predict outputs (i.e., motor behaviors). In our framework, the forward models run in parallel whereas in hierarchical predictive coding the internal generative models run in series and are interconnected by feedforward and feedback connections (Rao and Ballard, 1999). The feedforward ascending connections constitute an inverse model (i.e., using outputs to predict inputs) (Harth et al., 1987; Kawato et al., 1993; Friston, 2005). The difference between the output of a higher-level model and its input from a lower level model creates an error signal that is then used to modify the next input (which approximates using outputs to predict inputs). In contrast, the feedback descending connections represent a forward model (i.e., inputs from higher levels are used to generate output predictions of what caused the lower level inputs). Consequently, in hierarchical predictive coding, top-down predictions modulate bottom up processing. In our algorithm, bottom-up predictions lie outside of the causal chain of processing and hence are able to contribute to an inner (implicit) sense of awareness of what is being processing.

Forward models as used in our algorithm have been empirically tested and confirmed in movement performance by robots (Tani, 2016) and *in silico* models of artificial self-awareness (i.e., gambling; Cleeremans, 2011). These forward models differ from those used in hierarchical predictive coding frameworks to explain visual recognition (Rao and Ballard, 1999; Friston, 2005). These latter models rely on top-down inputs in a linear hierarchy to infer what is currently being processed whereas the models in our algorithm use bottom-up inputs that uniquely feed into parallel models. While the hierarchical top-down models can explain visual recognition and categorization (Friston and Kiebel, 2009), these models are an integral component of the processing pipeline and do not act as external state observers (**Figure 3**) of the type needed for the system to develop a sense of awareness of its neural processing states (Cleeremans, 2011; Dehaene et al., 2017).

Seth (2013) uses a hierarchy of top-down-driven forward models to explain emotional responses with the ultimate driver being higher-order goals. While his proposed framework is specifically aimed at accounting for motor responses he postulates that a conscious emotion (i.e., emotional awareness) arises from the integration of sensory predictions across multiple levels. Unfortunately this idea is not further interrogated in later explorations of the role of predictive coding and active interoceptive inference in emotions (Seth and Friston, 2016). Nonetheless, higher level integration (lying outside of the causal chain of emotional responses) is consistent with our idea that integration of predictions with other pertinent system inputs forms the basis of a higher-level of awareness necessary, but not sufficient, for the feeling of pain.

Like Seth (2013), Barrett (2017) also believes that hierarchical predictive coding uses internal, generative models to anticipate and make inferences about ongoing sensory stimuli and, hence, drive motor and visceromotor actions. In the theory of constructed emotion, Barrett (2017) proposes that when predictive coding is used to meaningfully categorize or conceptualize sensations (e.g., as happiness), then one consciously experiences that sensation (i.e., as happiness). For Barrett (2017) an affective conscious state somehow emerges when predictions are given conceptual meaning. Barrett (2017) does not make it clear, however, why conceptual meaning should feel like something rather than nothing. One could imagine that predictions could lead to inferences about a particular mental state, but there remains an explanatory gap with respects to how that state could possibly feel like something.

## Localization of Pain Awareness in the Human Brain

Our proposed algorithm involves at least three hierarchical levels (rather than two as proposed by the higher-order thought theory; cf. Rosenthal, 1993). The first level has the external stimulus as the object of intent (referred to as “sensory processing”). The second level has “sensory processing” as the object of intent (referred to as “sensory awareness”). This level is proposed to recognize that a particular type of sensory information is being processed. The third level has “sensory awareness” as its object of intent (referred to as “inner awareness”). By its ability to recognize that it is aware of some sensory stimulus this level has created an inner awareness of its internal processing.

There is some disagreement with respect to the cortical localization of conscious awareness in the human brain. First-order theorists contend that awareness directly arises in the earliest stages of cortical processing of sensory input (Dretske, 1993). Higher-order theorists instead subscribe to the idea that conscious experience only occurs when a higher-order of neural processing becomes aware of first-order sensory processing (Rosenthal, 1993). There is little neuroanatomical and neurophysiological support for conscious experience arising directly from first-order sensory processing. For instance, in the visual system, conscious awareness of color is dependent on processing occurring in V4, a higher-order cortical region (Gegenfurtner, 2003). Lesions in this cortical region lead to achromatopsia. Moreover, visual awareness is still present following direct stimulation of higher visual cortices in cortically blind subjects (due to lesions in their first-order V1 cortex) (Mazzi et al., 2014; Bagattini et al., 2015). Feedback from cortical areas higher than V1 is considered essential for visual awareness in normal sighted individuals (Lamme and Roelfsema, 2000; Pascual-Leone and Walsh, 2001; Hurme et al., 2017). However, the role of feedback from higher visual cortices remains unclear and continues to be investigated (Klink et al., 2017). It is important to note that these higher-order visual cortices might be necessary but not sufficient for visual awareness. Higher cortical non-visual posterior areas are also likely to be necessary for visual awareness (Koch et al., 2016).

Although there are problems with trying to correlate subjective experience of visual stimuli with evoked cortical potentials (Aru et al., 2012; Koch et al., 2016), it remains a valuable approach for providing some mechanistic insights into the localization of awareness (Koch et al., 2016). Visual awareness consistently correlates with a negative potential with onset at ∼200 ms and then a positive potential arising at ∼300 ms after stimulus presentation (Koivisto and Revonsuo, 2010; Rutiku et al., 2016; Rutiku and Bachmann, 2017). There is considerable variability between studies in the reported timing of these potentials but there is agreement that the negative potential (referred to as the visual awareness negativity) that occurs over the occipital-temporal-posterior parietal cortices is a signature of visual awareness (Koch et al., 2016; Schelonka et al., 2017).

Some of the temporal variability is associated with technical differences between studies. For instance, Schelonka et al. (2017) recorded the largest amplitude of the negative potential between 320 and 380 ms post-stimulus, whereas Rutiku et al. (2016) reported that the mean amplitude occurred at ∼240 ms and Shafto and Pitts (2015) timed the negative potential at ∼260–300 ms. Notwithstanding these differences, the timing of this potential is relatively late and not consistent with awareness arising solely from within the early visual cortex. The role of the occipital and posterior parietal cortices in visual awareness have also been highlighted by functional magnetic imaging studies during binocular rivalry with no-report paradigms (Frässle et al., 2014). We have avoided the controversial discussion of whether or not the prefrontal cortex is necessary for visual awareness (Safavi et al., 2014; Boly et al., 2017; Odegaard et al., 2017) since it is enough here to conclude that the primary visual cortex is not sufficient for awareness.

**FIGURE 5:**
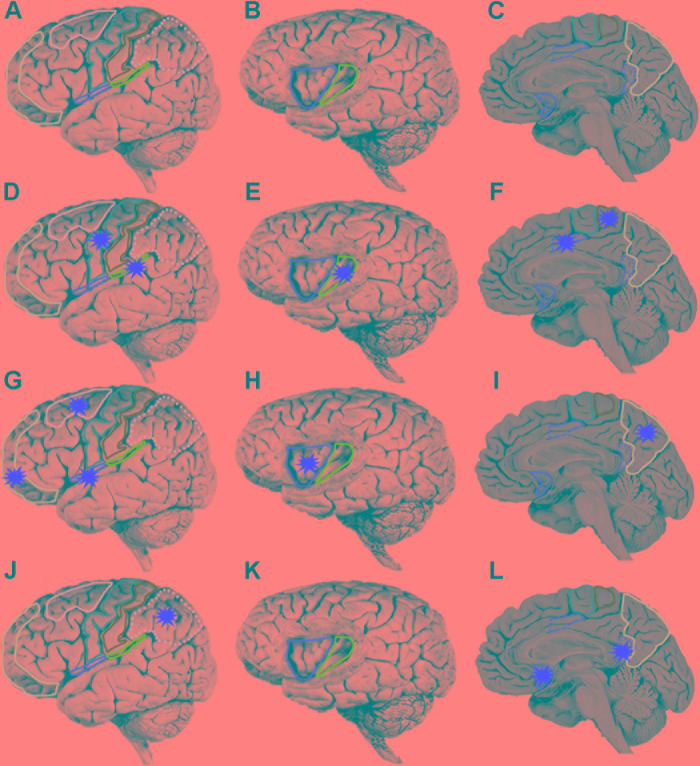
Localization of human brain regions. **(A,D,G,J)** Lateral view of brain, orbitofrontal cortex (mauve), dorsolateral prefrontal cortex (pink), motor cortex (purple), somatosensory cortex (green), posterior parietal cortex (dotted pink), lateral margin of frontal operculum (red), lateral margin of parietal operculum (blue). On a lateral view only the lateral margins of the operculum are exposed. The operculum forms the roof of the lateral sulcus and is only accessible by removal of the temporal lobe (which forms the ventral floor of the lateral sulcus). **(B,E,H,K)** Lateral view of brain with partial frontoparietal lobe removed to reveal underlying gyri of insula, anterior insula (red), posterior insula (green). **(C,F,I,L)** Medial wall of brain, perigenual anterior cingulate cortex (red), posterior mid cingulate cortex (dashed red), ventral posterior cingulate cortex (dotted red), supplementary motor area (purple), somatosensory cortex (green), and precuneus (pink). Each of the three rows starting at **(D,G,J)** represent a temporal sequence of cortical activation (red stars). **(D–F)** After initial activation of the somatosensory area there is co-activation of five cortical areas **(G–I)**. A third wave of co-activation of five cortical area. **(J–L)** A fourth wave of co-activation of three cortical areas.

Unlike the visual system, there is no primary cortical region for pain (Treede et al., 1999; Mano and Seymour, 2015; Thomas, 2017). Neuroimaging has revealed that a network of first-order and higher-order cortical regions including somatosensory areas I (SI) and II (SII), and insular and cingulate cortices is specifically activated during pain awareness in humans (**Figures 5A–C**; Rolls et al., 2003; Vogt, 2005; Wager et al., 2013; Vogt et al., 2016). This result is consistent with transneuronal pathway tracing experiments that demonstrated the terminations of the spinothalamic tract, the main ascending tract transmitting noxious information to the cortex, principally within the posterior insular, SII and cingulate cortices in monkeys (Dum et al., 2009). Similar pathways have been demonstrated by neuroimaging studies in humans (Brooks and Tracey, 2005; Omori et al., 2013). Human neurophysiological studies have also revealed nociceptive responses within SI, SII, insular and cingulate cortices (Frot et al., 2001, 2013, 2014; Liberati et al., 2017). Lesions and electrical stimulation in these cortical regions as well as strokes involving the spinothalamic tract in the internal capsule have together confirmed a role of this cortical network in human pain awareness (Ballantine et al., 1967; Berthier et al., 1988; Kim, 1992; Cereda et al., 2002; Torta et al., 2013; Boccard et al., 2014, 2017; Hirayama et al., 2014; Russo and Sheth, 2015; Agarwal et al., 2016; Denis et al., 2016; Wang et al., 2017).

Most human neuroimaging studies using functional magnetic resonance imaging are insensitive to dynamic temporal changes in neural activity across the cortical networks (Kucyi and Davis, 2015; Morton et al., 2016) and, hence, do not adequately reflect activity correlating with instances of pain awareness. Intracortical electroencephalograph recordings have instead provided more precise temporal resolution of cortical activation following noxious stimulation (although early studies were limited by the small number of electrodes). SII responses contralateral to the side of noxious heat stimulation to the wrist and hand occur initially at a peak latency of 140 ms (Frot et al., 1999; Frot et al., 2001). These SII responses were specifically associated with stimuli that elicited pain and were not recorded from other sites including hippocampus, amygdala, temporal pole, temporal neocortex, cingulate gyrus, and orbitofrontal cortex (Frot et al., 2001). The insular cortex responds to noxious stimuli ∼40 ms after the SII with an initial peak at 180 ms (Frot and Mauguière, 2003). This analysis was not able to resolve any differences in latency of these potentials along the posterior-anterior axis of the insula. Subsequent analyses revealed that the SII responses were more selective for stimuli that were below pain threshold or only mildly painful, whereas posterior insular cortex responses more fully reflected thermal noxious stimuli clearly above pain threshold (Frot et al., 2006). These studies suggested that the posterior insular cortex and SII play different roles in pain awareness.

Intracortical electroencephalograph recordings from multiple sites simultaneously in the cingulate cortex and SII has begun to provide a clearer understanding of the spatiotemporal relationships of neural activity during pain perception (Frot et al., 2008). SII and posterior middle cingulate cortex (pMCC) co-activate initially at around 120–140 ms and this is followed by later activity in the posterior insula at ∼180 ms post-stimulus. In a subsequent study, it was revealed that areas 1 and 2 in SI consistently elicited intracortically recorded neural responses consisting of four components at ∼102, 129, 140, and 190 ms latency to noxious stimuli (Frot et al., 2013). Simultaneous recordings revealed a 126 ms response in the supra-sylvian operculum (which included SII) and a ∼218 ms latency biphasic potential in the insular cortex. The primary motor cortex I (M1) also responded with a distinctive triphasic potential beginning at ∼116 ms post-stimulus. A picture was emerging whereby noxious stimuli leads to initial processing in the SI, followed by activity in the parietal operculum (containing SII) and pMCC and then slightly later in the insular cortex.

Temporal analysis of processing in the insular cortex elicited by a noxious stimulus revealed that biphasic evoked potentials occurred first in the two posterior insular gyri (at 212–221 ms) and then slightly later in the three anterior insular gyri (237–309 ms) (Frot et al., 2014). Given that the anterior insula only receives a very minor (if any) direct projection from the spinothalamic noxious pathway (Dum et al., 2009), it appears that the shortest latency evoked potentials are serially processed in the insula, passing from the posterior to the anterior insula (Frot et al., 2014). This interpretation is consistent with known direct connections between the posterior and anterior insula (Mesulam and Mufson, 1982; Almashaikhi et al., 2014).

Pain awareness following a noxious laser irradiation of the hand occurs in a broad window of 260–422 ms (mean 349 ms) post-stimulus (Bastuji et al., 2016). This latency was measured as the time from noxious stimulation to a voluntary motor response (finger lift) that signified sensation of pain. However, given that cortical stimulation of the primary motor cortex elicits a wrist motor response within 20 ms (Calancie et al., 1987; Amassian et al., 1989), cortical activity leading up to the experience of pain must occur before a 240–400 ms temporal window (with a mean of 329 ms). Bastuji et al. (2016) found three consistent waves of onset of cortical activity: first, activity (at ∼120 ms) was detected in the posterior insula, parietal operculum (SII), MCC and supplementary motor area; second, activity was followed shortly later (beginning at ∼140 ms) in the frontal operculum, precuneus (part of superior parietal lobe), anterior insula, orbitofrontal cortex and dorsolateral prefrontal cortex; and third, activity beginning at 149 ms in the posterior parietal cortex, ventral posterior cingulate cortex (vPCC) and perigenual anterior cingulate cortex (pACC).

It should be noted that Bastuji et al. (2016) did not distinguish between regions of the MCC although in an earlier study they demonstrated that it was the pMCC that was selectively activated by noxious stimuli (Frot et al., 2008). The highest peak activity in most of these regions was reached by 319 ms, which is prior to the time of awareness (at 329 ms). Only peak activities in vPCC and pACC occurred after pain onset (at ∼350 and 398 ms respectively). Despite differences in electrode placement and noxious stimulation between the various electroencephalographic studies described above, it is apparent that there is a specific temporal pattern of activation of cortical regions leading up to pain. First, there is early activity in SI; second, co-activation of the SII, posterior insular, pMCC, M1 and supplementary motor area (**Figures 5D–F**); third, co-activation of anterior insular, precuneus, frontal operculum, orbitofrontal cortex and dorsolateral prefrontal cortex (**Figures 5G–I**); and finally, activity in the posterior parietal cortex, vPCC and pACC (**Figures 5J–L**). Onset of activity in all of these regions is temporally consistent with them contributing to neural processing leading to pain awareness.

## Cortical Neural Circuits Supporting a Parallel Forward Models Algorithm

**FIGURE 6:**
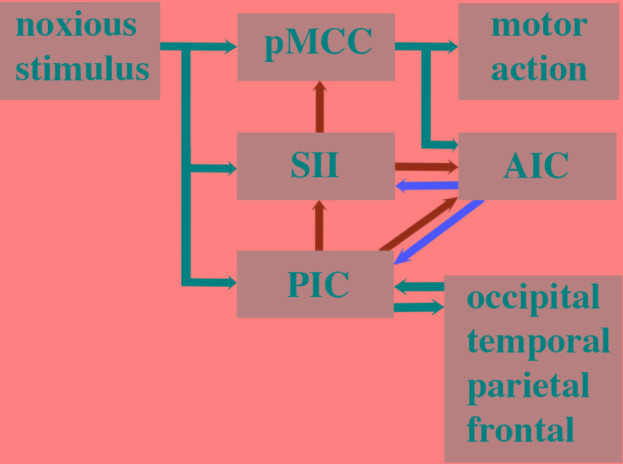
Human cortical circuitry consistent with the parallel forward models algorithm as presented in **Figure 4**. Parallel streams of noxious sensory input enter the posterior middle cingulate cortex (pMCC), secondary somatosensory area (SII), and posterior insula cortex. The SII and PIC contain internal models of pMCC and SII respectively. The outputs of these models are predictions (green arrows) and they are relayed to a comparator module in the anterior insular cortex (AIC). The AIC compares the output of pMCC and SII and the error (red arrow) is fed back to the SII to adjust its internal model of pMCC. The prediction of SII is fed back to the pMCC where it is used to refine its response to the noxious stimulus. The prediction of the internal model in PIC is fed forward to the AIC where it is compared with the prediction of SII. The error is then relayed back to the PIC to fine tune its internal model of SII. The prediction of the PIC is also fed back to the SII to refine its prediction. The internal model in the PIC is also adjusted in response to widespread feedback from across the cortex and the PIC has reciprocal connections with these regions that contribute to the feeling of pain.

The proposed parallel forward models algorithm (**Figure 4**) imposes structural restrictions on the types of nervous systems that could implement it. Is the structural connectivity captured by the algorithm neuroanatomically plausible? In the primate somatosensory system, the spinothalamic axon tract delivers sensory input from noxious stimuli into the cerebral cortex where it splits and terminates in three principal regions: cingulate cortex, SII and posterior insular cortex (PIC) (Dum et al., 2009). In humans, a main target in the cingulate cortex regulating motor responses (e.g., facial expressions, Kunz et al., 2011) to noxious stimuli is the pMCC (Perini and Bergstrand, 2013; Vogt et al., 2016). Each of these three regions were shown in electroencephalographic studies (discussed above) to be co-activated via noxious stimuli (**Figures 5D–F**) and by neural pathway tracing experiments to feed forward to the AIC (pMCC to AIC, Mufson and Mesulam, 1982; Mesulam and Mufson, 1982; SII to AIC, Mufson and Mesulam, 1982; Mesulam and Mufson, 1982; PIC to AIC, Almashaikhi et al., 2014). Both SII and PIC receive strong, reciprocal feedback from AIC (Mesulam and Mufson, 1982; Morecraft et al., 2015). PIC projects to SII (Mesulam and Mufson, 1985; Augustine, 1996; Cerliani et al., 2012), and SII in turn projects to pMCC (Morecraft et al., 2004; Vogt et al., 2005). This connectivity (**Figure 6**) is consistent with the pMCC processing sensory information to produce an output while putative internal models in SII and PIC create predictions that are compared in the AIC with the outputs of the pMCC and SII, respectively.

Somatosensory area II has previously been reported to play an important role in conscious awareness of somatosensory stimuli (Weisz et al., 2014), which is consistent with it participating in awareness of sensory processing in our algorithm. Strength of activity in SII has also been shown to be predictive for subsequent somatosensory awareness (Hirvonen and Palva, 2016), which also aligns to our framework. SII is additionally involved in distinguishing awareness of self-generated touch from external tactile stimuli (Blakemore et al., 1998). Taken together, the awareness arising in SII is consistent with this region generating predictions that are relayed to both AIC and pMCC where they are then available for modulating ongoing processing within this neural circuitry.

The prediction errors created in AIC are proposed to feed back to fine tune the performance of the internal models in PIC and SII (**Figure 6**). Functional neuroimaging studies (Metereau and Dreher, 2013; Allen et al., 2016; Meder et al., 2016; Bastin et al., 2017; Geuter et al., 2017) have confirmed a long-held view (Singer et al., 2009; Bossaerts, 2010; Ullsperger et al., 2010; Klein et al., 2013) that AIC is involved in error monitoring and awareness in humans. According to our algorithm, PIC is a higher order internal monitor that generates the awareness of awareness of sensory processing leading to motor behavior. Neuroimaging evidence and lesion data support such a role for PIC in awareness of limb position (Karnath et al., 2005) and self-awareness of motor actions (Baier and Karnath, 2008). We do not contend that predictions (awareness) and prediction error related to pain do not occur elsewhere in the brain (Ploghaus et al., 2000; Roy et al., 2014) but rather that such processing is important in modulating PIC predictions. The widespread connectivity of PIC (**Figure 6**) (Nomi et al., 2017) and its multimodal processing of visual, tactile, nociceptive, and vestibular information (zu Eulenburg et al., 2013; Frank et al., 2016) strengthen the proposal that its predictions need to be modulated by global input in order to ensure awareness functions for the whole system.

The proposed cortical neural circuitry underlying our algorithm (**Figure 6**) is supported by considerable evidence that pain is elicited by electrical stimulation and perturbed by lesions to the operculoinsular region (i.e., SII, AIC, and PIC; Bassetti et al., 1993; Cereda et al., 2002; Bowsher et al., 2004; Birklein et al., 2005; Cattaneo et al., 2007; Afif et al., 2008, 2010; Garcia-Larrea et al., 2010; Isnard et al., 2011; Mazzola et al., 2011, 2017; Hirayama et al., 2014; Montavont et al., 2015; Denis et al., 2016; Maesawa et al., 2016; Bouthillier and Nguyen, 2017; Garcia-Larrea and Bastuji, 2018; Garcia-Larrea and Mauguière, 2018). An example of one patient (referred to as Roger) with extensive bilateral damage to the insula who exhibited no deficits in pain sensation has been used to argue against a role of this brain region in pain (Feinstein et al., 2016). However, this case report needs to be considered in the context of the overwhelming evidence that the insula does play a key role in human pain. Negative results are difficult to interpret and can be explained by inter-subject variability in lesion site and size and post-lesion cortical plasticity (Key, 2016). For instance, Roger still had 22% of his left insula intact as well as the entire left SII and both left and right PCC (whether this included the pMCC was not assessed) and his lesion occurred about 30 years prior to sensory testing (Feinstein et al., 2010; Philippi et al., 2012).

Damasio et al. (2012) discuss another patient (patient B) with extensive cortical lesions involving the insula whom continued to experience pain. However, patient B had normal right and left SII, normal right MCC and only partially damaged left MCC. Unfortunately there was no high resolution or quantitative analysis of the lesion sites, no quantitative nociceptive sensory testing and no functional magnetic resonance imaging of patient B in response to nociceptive stimuli which makes interpretation of this case report problematic. Damasio et al. (2012) acknowledged themselves that pain could have been generated by a combination of undamaged cortical regions and cortical plasticity of function in patient B. This is an important point given that we are not contending the necessity of insula for pain but merely using evidence of the role of a neural circuit involving the insula to support the implementation of our algorithm in the human brain. It is entirely feasible that other cortical regions have internal models and comparator modules that can implement our algorithm (provided they possess the necessary neural interconnectivity; **Figure 6**).

## Cephalopods Lack the Neural Circuitry for Pain

**FIGURE 7:**
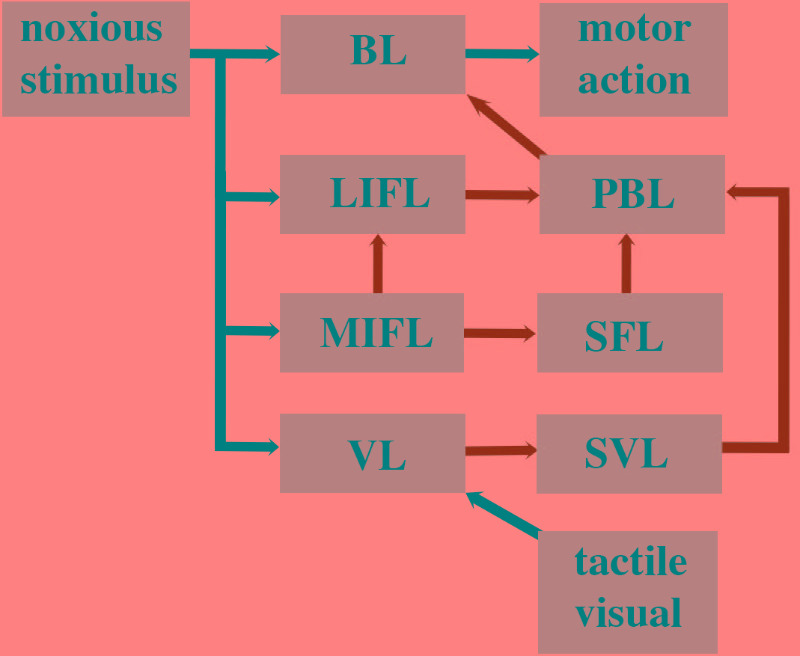
Octopus brain circuitry underlying processing of noxious stimuli based on Young (1991). The circuitry as described in **Figure 2** is schematized here to fit the parallel forward models algorithm. The circuitry can be compared with the human cortical circuitry presented in **Figure 6**. Noxious information is relayed in parallel to the brachial lobe (BL), lateral inferior frontal lobe (LIFL), medial inferior frontal lobe (MIFL), and the vertical lobe (VL). The VL feeds forward to the subvertical lobe (SVL). The MIFL feeds forward to the subfrontal lobe (SFL) and the LIFL. The SVL, SFL, and LIFL all feed forward to the posterior buccal lobe (PBL) which then feeds forward to the brachial lobe. The brachial lobe controls motor actions. The VL also receives input from the tactile and visual system.

Given that avoidance learning and neural lesioning approaches involving noxious stimuli are not evidence for pain, we next examined whether the cephalopod brain possessed the prerequisite neural circuitry as outlined in the parallel forward models algorithm (**Figures 5**, **6**). The basic circuitry in the supraesophageal brain underlying avoidance learning using noxious electrical shock during tactile discrimination was presented in **Figure 2**. When the subesophageal brachial lobe is included in this circuitry (**Figure 7**), it reveals that the noxious stimuli enter four parallel streams (which is consistent with our algorithm). The basal stream is from the arms into the brachial lobe and its output drives motor behavior. Noxious input also directly enters both the lateral and median inferior frontal lobes as well as the vertical lobe. Each of these lobes feeds forward to the posterior buccal lobe, which, in turn feeds forward to the brachial lobe to modulate motor actions. The vertical lobe receives global input from the tactile and visual systems. While the circuitry associated with noxious stimuli has some of the components as detailed in the parallel forward models algorithm, it critically lacks the necessary feedforward and feedback pathways between the lobes (cf. **Figure 6**). If the lateral and median inferior frontal lobes and vertical lobes were to generate predictions that were fed forward to the posterior buccal lobe, this latter lobe lacks the ability to feedback prediction errors to these lobes so as to regulate their models. The overall circuitry is instead consistent with a simple feedforward model that modulates motor outputs from the brachial lobe.

## Conclusion

We have argued here that behavioral responses to noxious stimuli in animals cannot be used to assess whether an animal feels pain. Attempting to reconcile behavioral responses to noxious stimuli with brain lesioning approaches leads to paradoxical conclusions about the origins of pain in the octopus brain. The experiment findings are instead congruous with octopi responding non-consciously to noxious stimuli. We contend that for any animal to feel pain it must possess the appropriate neural circuitry to perform the neural processing necessary for pain. Most extant models of consciousness have not specifically addressed the neural basis of feelings such as pain and have rather concentrated on recognition and discrimination in sensory perception. We have postulated here that feeling states are dependent on specific neural computations. Rather than reverse engineer the human brain in order to define these computations we have instead used basic design principles to construct an algorithm that forms a necessary although not sufficient basis for pain. Our framework is built on the premise that for any nervous system to be capable of feeling it must have the potential to be aware of changes in its own neural states. Although awareness begins first with detection of those changes it must also involve higher levels of awareness whereby the system becomes aware that it has detected those changes. We suggest that dedicated neural circuits (called state observers) must initially monitor the noxious sensory processing that generate motor behaviors. These state observers are themselves further monitored by additional state observers and this tiered circuitry leads to awareness of awareness of sensory processing – the fundamental neural basis of a feeling state.

Our algorithm is consistent with the widely held view that feelings have a functionally significant role for the organism. Such a role emerges naturally when the state observer creates an internal forward model that predicts the output of the neural processing based on its current input. That prediction is a cogent test of the system’s awareness of its ongoing processing. It also plays a functional role by its ability to modulate that same processing. We utilize an algorithm that incorporates parallel forward models that make predictions which are subsequently compared with future outputs. Differences between predictions and real outputs (prediction errors) are then used to train the forward models to become more accurate in their predictions. Those predictions are also used to bias sensory neural processing toward the predictions of the model and hence enhance efficacy of the neural processing. We show that the human brain possesses the necessary cortical circuitry to implement the algorithm. Further, we find that the octopus brain cannot execute this algorithm since it lacks the necessary feedforward and feedback pathways between brain regions associated with sensory processing of noxious stimuli. This inability to create an awareness of awareness of sensory processing is not consistent with the octopus feeling pain.

## Author Contributions

BK and DB contributed to the research and writing of this manuscript.

## Conflict of Interest Statement

The authors declare that the research was conducted in the absence of any commercial or financial relationships that could be construed as a potential conflict of interest. The reviewer GP and handling Editor declared their shared affiliation at the time of the review.
